# Gas concentration prediction based on SSA algorithm with CNN-BiLSTM-attention

**DOI:** 10.1038/s41598-025-15838-4

**Published:** 2025-10-01

**Authors:** Wenjing Xing, Yanguo Yang, Yanxin Zhang, Yong Yang

**Affiliations:** College of Safety and Emergency Management Engineering, Taiyuan University of Technology, Mingxiang Campus, Yuci District, Jinzhong, 030600 Shanxi Province China

**Keywords:** Gas concentration, Concentration prediction, Deep learning, Attention mechanism, Sparrow optimization algorithm, Environmental sciences, Energy science and technology, Engineering, Mathematics and computing

## Abstract

Accurate prediction of coal mine gas concentration is a crucial prerequisite for preventing gas exceed and disasters. However, the existing methods still suffer from issues such as low data utilization, difficulty in effectively integrating multivariate nonlinear spatiotemporal features, and poor generalization capability when achieving relatively high prediction accuracy but requiring longer prediction durations. To address these challenges, this study focuses on a tunneling face in a Shanxi coal mine and proposes a novel hybrid deep learning model (CNN-BiLSTM-Attention). The model employs a 1D-CNN to extract local spatial features of gas concentration, temperature, wind speed, rock pressure, and CO concentration, utilizes BiLSTM to model bidirectional temporal dependencies, and incorporates an attention mechanism to dynamically weight critical features, such as sudden changes in gas concentration. Additionally, the sparserow search algorithm (SSA) was applied to automatically optimize hyperparameters, including the number of CNN filters and BiLSTM hidden units. The results demonstrate that the proposed SCBA model achieves an RMSE of 0.0171 and MAPE of 0.084. Compared to mainstream models such as attention-LSTM, SSA-LSTM-aAttention, and rTransformer-LSTM, the RMSE was improved by 23.3%, 4.4%, and 30.2%, respectively, while the MAPE was improved by 38.7%, 14.3%, and 43.62%, respectively, indicating superior prediction accuracy. To validate the contribution of each module, ablation experiments were conducted by sequentially removing the CNN, BiLSTM, and Attention components for error analysis, confirming the necessity of the multi-module collaborative mechanism. In multi-step predictions, the proposed model exhibits better generalization capability as the prediction horizon increases, with the slowest error growth, providing insights for enhancing gas concentration prediction generalization. The model achieves the smallest error at 20 time steps, laying a foundation for subsequent anomaly analysis and offering miners sufficient safety evacuation time, thereby establishing a reliable basis for real-time gas disaster early warning.

## Introduction

Methane gas represents a significant hazard in underground mining operations, where its concentration dynamics critically impact mine safety^1^. Consequently, accurate prediction of gas concentration trends is essential for implementing timely safety interventions, protecting miners’ lives, and preventing catastrophic gas-related incidents. The integration of artificial intelligence and machine learning techniques in coal mining has elevated precise gas concentration forecasting to a paramount research priority in mine safety management, particularly for preventing hazardous gas level exceedances^2^.

Conventional gas prediction methodologies predominantly employ two approaches: statistical modeling and physics-based simulation^3^. Statistical techniques, including Autoregressive Integrated Moving Average (ARIMA) models^4^ and multiple linear regression^5,6^, demonstrate effectiveness with limited datasets but necessitate manual recalibration for maintaining predictive performance. Physics-driven approaches, such as turbulent diffusion Eq^7^. and computational fluid dynamics models^8^, provide system-level characterization of gas dispersion patterns through first-principles modeling. While valuable for comprehensive mine gas distribution analysis, these physical models exhibit limitations in long-term forecasting and high-sensitivity detection scenarios.

Due to the limitations of traditional methods in handling complex mining environments and nonlinear data, machine learning approaches^9^ have been widely adopted in predictive applications. For instance, Support Vector Machines (SVM) have been employed for river flow prediction^10^, while Backpropagation (BP) neural networks have been applied in biological studies to predict protein content^11^. In the domain of gas concentration prediction, Xu et al.^12^ proposed methods based on machine learning algorithms including random forests and Gradient Boosting Decision Trees (GBDT). These approaches effectively address issues such as low regression prediction accuracy, thereby significantly improving prediction precision.

Several studies have enhanced gas concentration prediction accuracy by employing optimization algorithms to tune model parameters. For example, Particle Swarm Optimization (PSO)^13^ and Genetic Algorithms (GA)^14^ have been utilized to optimize the penalty and kernel parameters of SVM. Representative applications include GA-optimized SVM (GA-SVM)^15,16^, Grey Wolf Optimizer-based SVM (GWO-SVM)^17^, and Sparrow Search Algorithm-optimized SVM (SSA-SVM)^18^. These optimization algorithm-enhanced machine learning methods have demonstrated significant performance improvements in prediction tasks.

While the traditional machine learning approaches exhibit limitations, deep learning has emerged as a superior alternative for gas concentration prediction, owing to its exceptional feature extraction and temporal modeling capabilities^19^. A representative advancement is the evolutionary attention-based temporal graph convolutional network proposed by Cheng et al.^20^, which effectively captures spatiotemporal correlations in gas concentration data, leading to significant accuracy improvements.

Convolutional Neural Networks (CNNs), renowned for their success in image processing^21^ and time-series analysis^22^, demonstrate particular efficacy in extracting spatial features from multidimensional gas sensor data^23^. Nevertheless, gas concentration datasets inherently possess strong temporal characteristics, as concentrations dynamically respond to evolving mining conditions. This temporal dependency has motivated the adoption of Long Short-Term Memory (LSTM) networks^24^, which excel at modeling long-range dependencies in time-series data. For instance, Xu et al.^25^ developed an enhanced LSTM framework incorporating an improved whale optimization algorithm, achieving notable prediction accuracy gains.

Recent advances have witnessed the emergence of hybrid CNN-LSTM architectures that synergistically capture both spatial and temporal features, demonstrating superior performance across diverse prediction tasks^26,27^. The integration of self-attention mechanisms^28^ further enhances these models by dynamically weighting features across temporal steps, significantly improving prediction accuracy - particularly for complex dependency patterns. This architecture proves especially effective in identifying critical temporal features, such as abrupt gas concentration fluctuations that demand immediate attention. Notwithstanding these advancements, current gas concentration prediction systems still face three fundamental limitations: First, existing approaches inadequately exploit latent data features and fail to effectively integrate multidimensional environmental variables for comprehensive prediction. Second, the predominant reliance on conventional optimization algorithms leads to suboptimal parameter tuning, characterized by inefficient convergence and susceptibility to local optima. Third, while achieving satisfactory accuracy, most models remain constrained to short-term forecasting windows. This proves particularly inadequate in high-risk mining scenarios where regulatory requirements mandate at least 20 min of advance warning - the critical timeframe necessary for miner evacuation during tunneling operations.

To address these challenges, this study proposes a novel gas concentration prediction framework that integrates Sparrow Search Algorithm (SSA)-optimized CNN-BiLSTM with an attention mechanism. The proposed model employs a three-tier architecture: (1) a Bidirectional LSTM (BiLSTM) network to comprehensively capture both forward and backward temporal dependencies, (2) Convolutional Neural Networks (CNN) to extract localized spatial correlations from multivariate environmental factors, and (3) an attention mechanism to dynamically prioritize critical temporal patterns, particularly abrupt gas concentration variations.

The SSA optimization algorithm was implemented to systematically tune model hyperparameters, thereby enhancing both prediction accuracy and stability. Furthermore, the framework incorporates multidimensional feature fusion by jointly analyzing gas concentration data with key environmental variables - including temperature, wind speed, rock pressure, and CO concentration - enabling the model to: (i) preserve intrinsic gas concentration dynamics while (ii) effectively modeling environmental influence factors. This integrated approach achieves superior performance by simultaneously considering spatial-temporal dependencies and environmental covariate effects.

## Basic theoretical methods

The underground mining environment exhibits complex and dynamic characteristics^29^. Gas concentration monitoring data - influenced by multiple coupled factors (including geological structures, mining processes, and ventilation conditions) - demonstrate both significant nonlinear dynamics and non-stationary time-series features. To address these challenges in forecasting high-dimensional, noisy time-series data, this study proposes a prediction model incorporating intelligent optimization algorithms to enhance gas concentration prediction accuracy.

### Bidirectional long short-term memory network (BiLSTM)

Bidirectional long short-term memory networks (BiLSTM) represent a class of deep learning models that have been extensively applied across multiple domains, including natural language processing, speech recognition, and time series forecasting^30^. Developed to overcome the limitations of conventional unidirectional LSTMs (which process information in only the temporal-forward direction), BiLSTM architectures incorporate two complementary LSTM layers:

Forward LSTM: Processes sequences from beginning to end, capturing historical patterns;

Backward LSTM: Processes sequences from end to beginning, extracting subsequent contextual information.

1 $$\:\overrightarrow{{h}_{\text{t}}}=\text{L}\text{S}\text{T}\text{M}\left({\text{x}}_{\text{t}},{\overrightarrow{h}}_{\text{t}-1}\right)$$

2 $$\:{\overleftarrow{h}}_{\text{t}}=\text{L}\text{S}\text{T}\text{M}\left({\text{x}}_{\text{t}},{\overleftarrow{h}}_{\text{t}-1}\right)$$

3 $$\:{\text{y}}_{\text{t}}=\overrightarrow{\text{W}}\overrightarrow{{h}_{\text{t}}}+\overleftarrow{\text{W}}{\overleftarrow{h}}_{\text{t}}+{\text{b}}_{\text{y}}$$

**Fig. 1:**
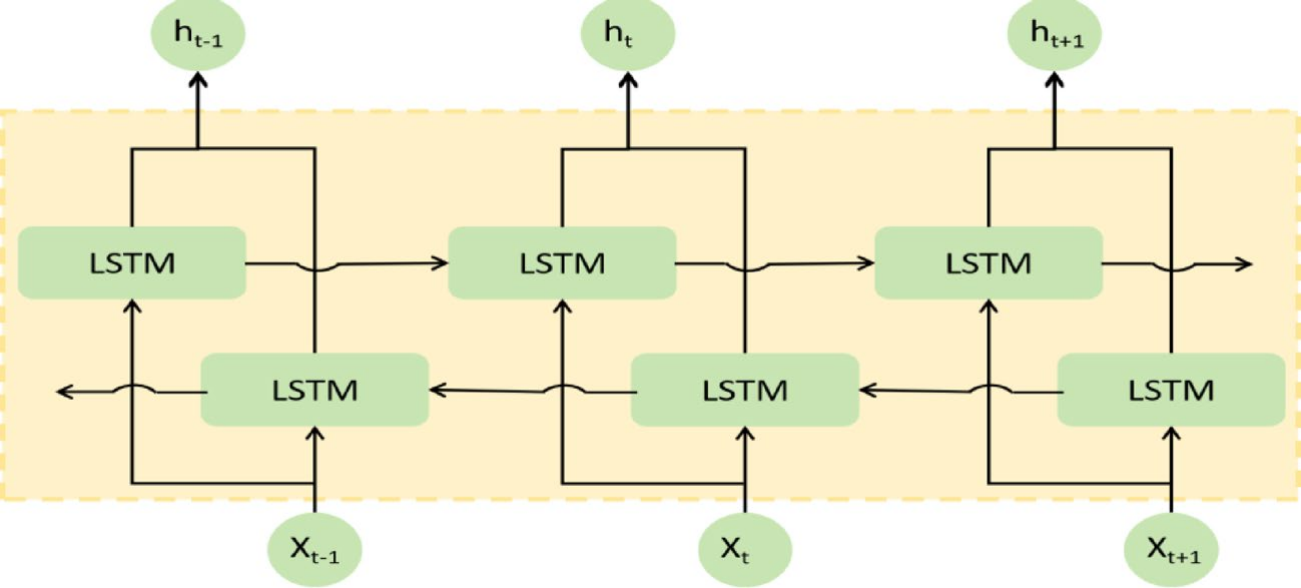
BiLSTM structure diagram.

By combining the forward and backward LSTM layers, BiLSTM fully captures temporal dependencies in the data, improving the prediction accuracy and robustness—especially when dealing with complex time series forecasting tasks^31^.The BiLSTM structure is shown in Fig. 1.

### Convolutional neural networks (CNN) and attention mechanism

Convolutional Neural Networks (CNNs) are deep feedforward networks specifically designed to process grid-like data (such as images and time series)^32^. They automatically extract local features through convolution operations, significantly reducing the number of parameters and enhancing the feature representation capability. In gas concentration prediction, CNNs excel in capturing local abrupt patterns and periodic fluctuations in time series data.

**Fig. 2:**
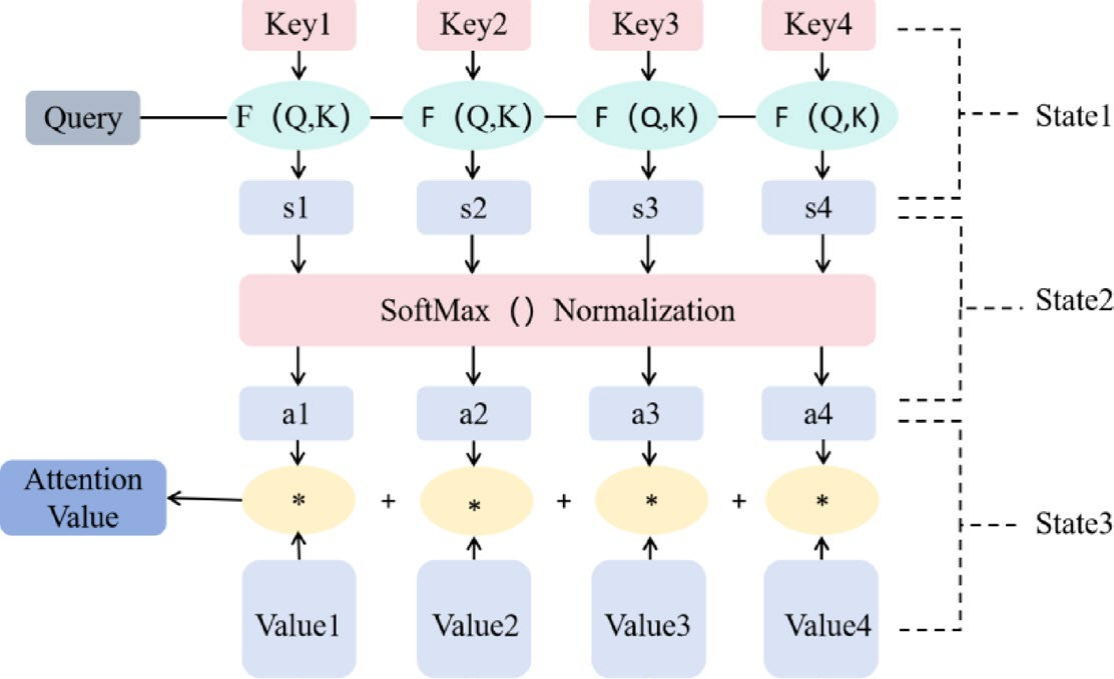
Attention structure diagram figure.

The attention mechanism is a computational model that simulates human attention selection ability. When processing information, it allows the model to focus on specific parts of the input data, thereby increasing the weight of key information while ignoring irrelevant data^33^. Essentially, it is a weighted summation process that assigns different weight values to different input data, altering the model’s attention to the data and making the model output more accurate. The attention mechanism shifts focus from considering everything to focusing on parts of the input.The structure of the attention mechanism is shown in Fig. 2.

In the field of time series forecasting, the attention mechanism enhances model prediction performance by: (1)Highlighting key features strongly correlated with long-term predictions. (2)Suppressing irrelevant information. This selective focus enables more efficient allocation of computational resources to the most significant features.

### Sparrow search algorithm (SSA)

The Sparrow Search Algorithm (SSA) was proposed in 2020 as a novel population optimization algorithm inspired by sparrows’ foraging and anti-predation behaviors^34^. By simulating these mechanisms, it divides populations into three roles: Discoverers (high-fitness individuals who locate food first), Followers, and Vigilants.

Discoverers, having access to larger food ranges, provide foraging guidance for the entire population.

The position update formula is:

4 $$\:{\text{X}}_{\text{i},\text{j}}^{\text{t}+1}=\left\{\begin{array}{c}{\text{X}}_{\text{i},\text{j}}^{\text{t}}\cdot\:exp\left(\frac{-\text{i}}{\cdot\:{\text{i}\text{t}\text{e}\text{r}}_{\text{m}\text{a}\text{x}}}\right),{\text{R}}_{2}<ST\\\:{\text{X}}_{\text{i},\text{j}}^{\text{t}}+Q\cdot\:L,{\text{R}}_{2}\ge\:ST\end{array}\right.$$

where: t represents the iteration count, the position of the i-th individual in the j-th dimension at the t-th iteration as $\:{\text{X}}_{\text{i},\text{j}}^{\text{t}}$; R_2_∈ [0,1] is the alarm value, and ST ∈ [0.5,1] is the safety threshold.

If R_2_ < the population is in a safe state, and there are no predators in the surrounding environment. Discoverers can expand their search range.

If R_2_ > safety threshold, a predator is detected by an individual and an alarm is triggered. The discoverer must lead the population into a safe foraging area.

The position update formula for discoverers is:

5 $$\:{\text{X}}_{\text{i},\text{j}}^{\text{t}+1}=\left\{\begin{array}{c}Q\cdot\:exp\left(\frac{{\text{X}}_{\text{w}\text{o}\text{r}\text{s}\text{t}}^{\text{t}}-{\text{X}}_{\text{i},\text{j}}^{\text{t}}}{{\upalpha\:}\cdot\:{\text{i}\text{t}\text{e}\text{r}}_{\text{m}\text{a}\text{x}}}\right),i>n/2\\\:{\text{X}}_{\text{P}}^{\text{t}+1}+\left|{\text{X}}_{\text{i},\text{j}}^{\text{t}}-{\text{X}}_{\text{P}}^{\text{t}+1}\right|\cdot\:{\text{A}}^{+},otherwise\end{array}\right.$$

where:$\:{\text{X}}_{\text{P}}^{\text{t}+1}$ is the current individual’s optimal position; $\:{\text{X}}_{\text{w}\text{o}\text{r}\text{s}\text{t}}^{\text{t}}$ is the global worst position from the last iteration.

When i > n/2, the fitness value of the i-th individual is low, and no food is found, requiring a position shift.

When the population converges to a local optimum, vigilant individuals are activated to perform random movements, thereby enhancing population diversity. These vigilant individuals typically constitute 10–20% of the total population. Their position update formula is defined as:

6 $$\:{\text{X}}_{\text{i},\text{j}}^{\text{t}+1}=\left\{\begin{array}{c}{\text{X}}_{\text{b}\text{e}\text{s}\text{t}}^{\text{t}}+\beta\:\cdot\:\left|{\text{X}}_{\text{i},\text{j}}^{\text{t}}-{\text{X}}_{\text{b}\text{e}\text{s}\text{t}}^{\text{t}}\right|,{\text{f}}_{\text{i}}>{\text{f}}_{\text{g}}\\\:{\text{X}}_{\text{i}.\text{j}}^{\text{t}}+K\cdot\:\left(\frac{\left|{\text{X}}_{\text{i},\text{j}}^{\text{t}}-{\text{X}}_{\text{w}\text{o}\text{r}\text{s}\text{t}}^{\text{t}}\right|}{\left({\text{f}}_{\text{i}}-{\text{f}}_{\text{w}}\right)+{\upepsilon\:}}\right),{\text{f}}_{\text{i}}={\text{f}}_{\text{g}}\end{array}\right.$$

where:$\:{\text{X}}_{\text{b}\text{e}\text{s}\text{t}}^{\text{t}}$ is the current best position of the entire population; K ∈ [−1, 1]; f_i_ is the current individual’s fitness value; fw and fg are the global worst and best fitness values, respectively.

When f_i_ > fg, it indicates that the individual is at the periphery of the safe zone and is prone to predator attacks.

When f_i_ = fg, it means that an individual located at the center of the safe zone has detected a predator and needs to move towards nearby individuals to reduce the likelihood of being attacked.

### Gas concentration prediction model construction

#### CNN-BiLSTM-attention model construction

The CNN-BiLSTM-Attention model architecture comprises seven key components: (1) input layer, (2) CNN layer, (3) dropout layer, (4) BiLSTM layer, (5) connection layer, (6) attention mechanism, and (7) fully connected layer.

The input layer receives gas concentration data and other influencing factors as inputs. Within the CNN component:

The convolutional layer performs spatial feature extraction via convolution operations.

The pooling layer reduces feature dimensionality while preserving critical information.

The dropout layer randomly deactivates features to prevent overfitting.

**Fig. 3:**
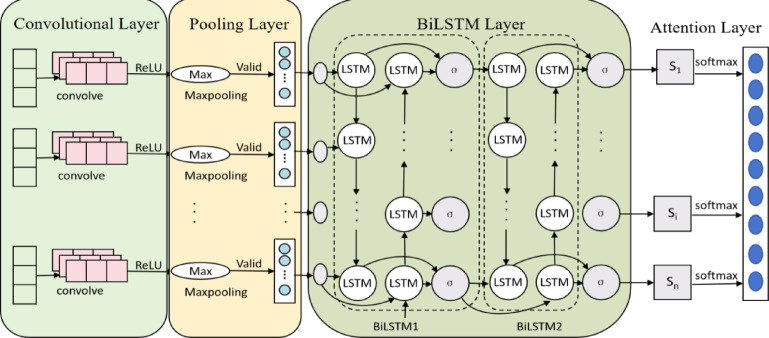
CNN-BiLSTM-Attention Structure Diagram.

Processed features are then propagated to the BiLSTM layer, which analyzes temporal dependencies through bidirectional processing (forward and backward directions). The connection layer subsequently merges these bidirectional outputs and feeds them into the attention mechanism. This attention layer implements dynamic feature weighting, emphasizing diagnostically relevant features at key timesteps. The architecture ultimately generates gas concentration predictions through the fully connected layer.The structure of CNN-BiLSTM-Attention is shown in Fig. 3.

#### Prediction model with sparrow search algorithm

To further improve the model’s performance and accuracy, a sparrow search algorithm (SSA) was introduced for hyperparameter optimization^34^. The Sparrow Search Algorithm is an optimization technique inspired by the foraging behavior of sparrows. By balancing exploration and exploitation, it can effectively search for an optimal solution. By optimizing hyperparameters (such as CNN kernel size, BiLSTM layer units, and attention weights), SSA can automatically adjust the hyperparameter settings in the model structure, thereby enhancing the model’s learning capability and prediction accuracy, and further improving the robustness and accuracy of gas concentration predictions.

The steps for building the gas prediction model based on SSA-optimized CNN-BiLSTM-attention are as follows:

Step 1: Preprocess the coal mine monitoring time series data, including handling missing values, outliers, noise, impact factor analysis, and normalization, then split the data into training and testing sets according to a specified ratio.

Step 2: Set the relevant parameters for the Sparrow Search Algorithm (SSA), such as the maximum number of iterations and population size. Configure the BiLSTM hidden layer units, attention mechanism weights, and the number and size of CNN kernels, then randomize the initial population.

Step 3: Build the CNN-BiLSTM-Attention neural network model.

Step 4: Use the Sparrow Search Algorithm (SSA) to optimize the hyperparameters. Update each sparrow individual’s position according to the SSA position update rules. Each individual represents a hyperparameter combination (e.g., LSTM units and learning rate). Through position updates, individuals move toward the global optimal solution while recording optimal values. During optimization, gradually reduce the search space using a predefined nonlinear shrinkage factor to enhance local search ability and accelerate convergence.

Step 5: Check whether the stopping conditions (maximum iterations or minimum criteria) are met. If satisfied, stop optimization, record the optimal hyperparameter combination, and assign it to the BiLSTM. Otherwise, return to Step 3.

Step 6: Build the final CNN-BiLSTM-Attention multiparameter gas concentration prediction model using the SSA-optimized hyperparameters, output multistep predictions, and perform inverse normalization on the results.

**Fig. 4:**
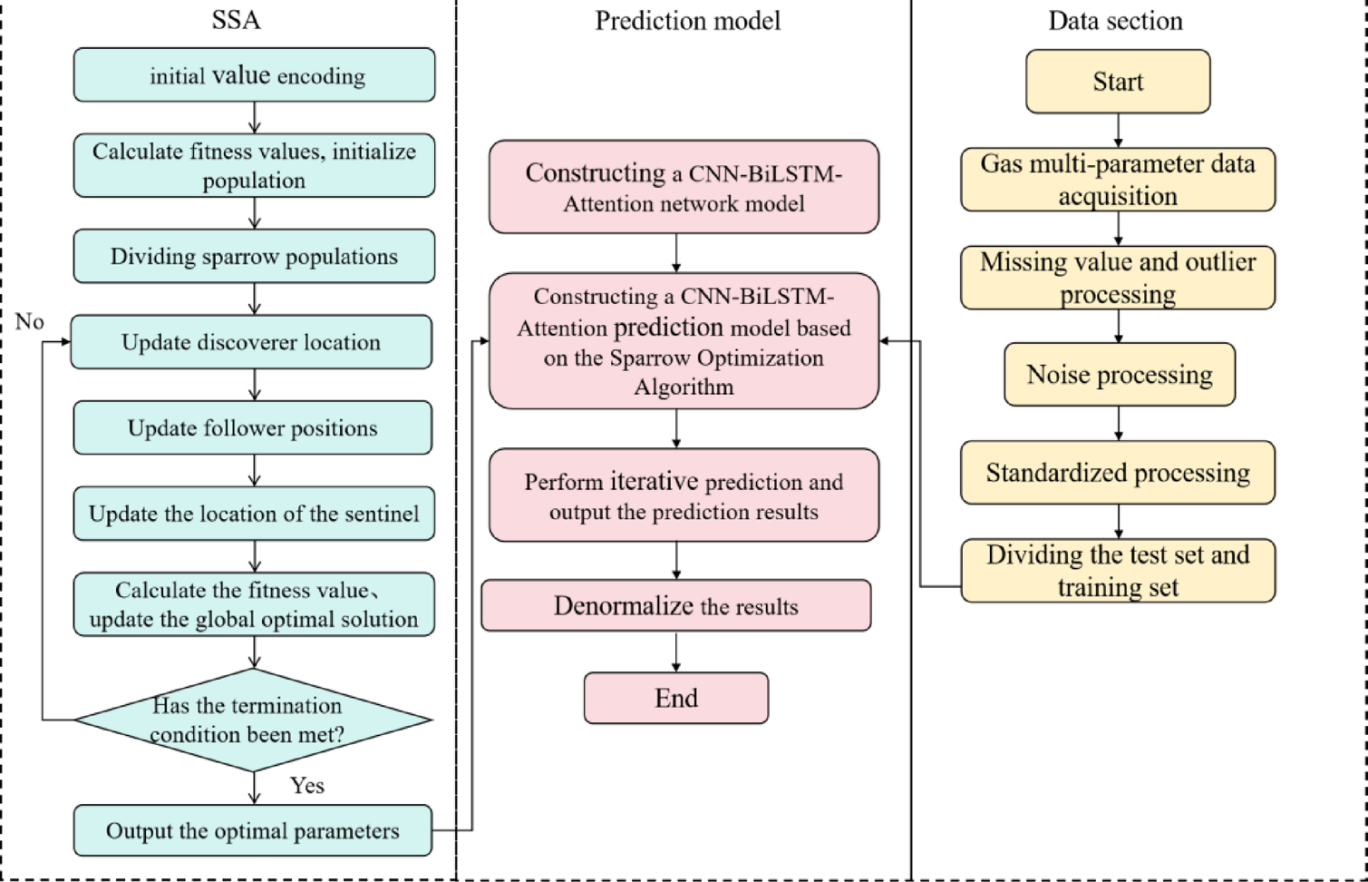
SSA for CNN-BiLSTM-Attention flow chart.

The optimization process of SSA for the CNN-BiLSTM-Attention structure is shown in Fig. 4.

#### Model performance evaluation

Gas concentration time series prediction is essentially a regression problem. To validate the prediction model performance, the Mean Absolute Percentage Error (MAPE) and Root Mean Squared Error (RMSE) were selected as evaluation metrics.

The Mean Absolute Percentage Error (MAPE) represents the average percentage of relative error between predicted and actual values. The calculation formula is:

7 $$\:\text{M}\text{A}\text{P}\text{E}=\frac{1}{\text{n}}\sum\:_{\text{i}=1}^{\text{n}}\left|\frac{\widehat{{\text{y}}_{\text{i}}}-{\text{y}}_{\text{i}}}{{\text{y}}_{\text{i}}}\right|$$

The Root Mean Squared Error (RMSE) measures the deviation between predicted values and actual observations. As a squared metric, it is sensitive to outliers and reflects prediction error magnitude:

8 $$\:\text{R}\text{M}\text{S}\text{E}=\sqrt{\frac{1}{\text{m}}\sum\:_{\text{i}=1}^{\text{m}}{\left({\text{y}}_{\text{i}}-\widehat{{\text{y}}_{\text{i}}}\right)}^{2}}$$

Where: n is the sample size, y_i_ is the actual value of the ith sample, and $\:\widehat{{\text{y}}_{\text{i}}}$ is the predicted value of the ith sample.

## Results and discussion

### Data collection and preprocessing

The sample data for this experiment were collected from the daily monitoring data of a mining face in Shanxi Province. The data includes five indicators: gas concentration (D1), temperature (D2), wind speed (D3), mine pressure (D4), and carbon monoxide (CO) concentration (D5). Data was collected every minute for a total of 2,880 data points. The training and test sets were split in an 80:20 ratio. The first 2,304 data points were used for model training, with 70% of the training set used for training and 10% used for validation. The remaining 576 data points were used to test the model’s prediction performance.

**Fig. 5:**
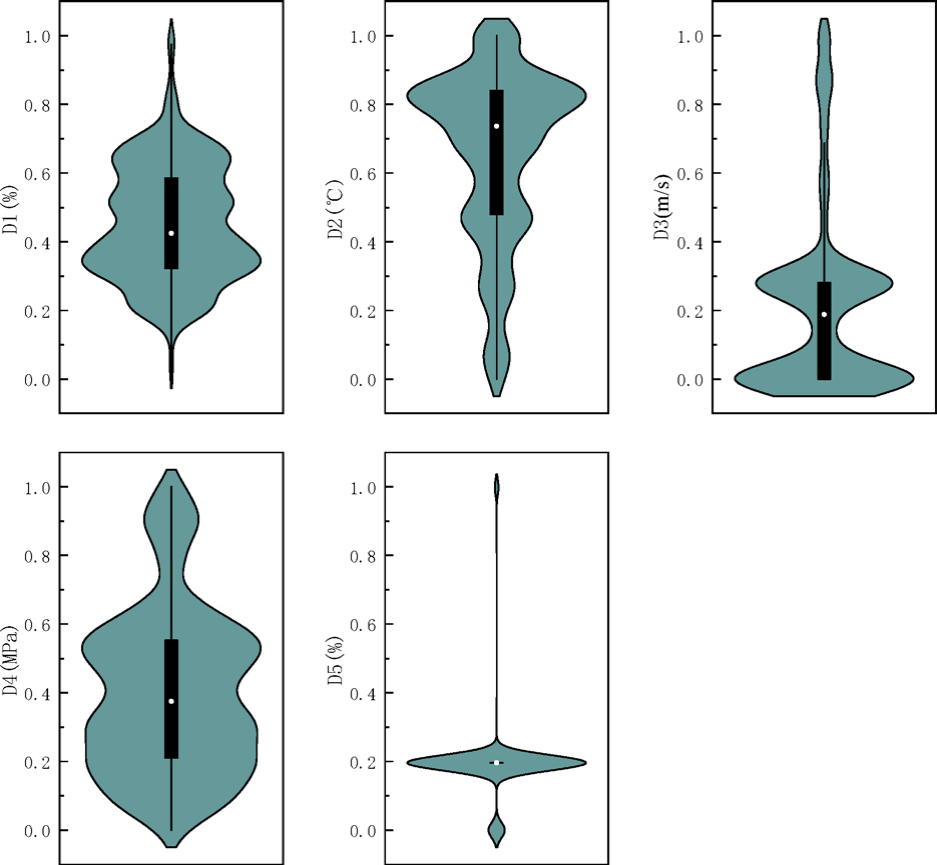
Normalized data distribution after preprocessing.

The normalized data distribution violin plot after applying wavelet denoising and cubic exponential smoothing for missing value treatment is shown in Fig. 5. From the plot, it can be observed that after preprocessing, the data distribution of each indicator was relatively concentrated, with fewer outliers, indicating good data quality.

The Pearson coefficient was used to assess the linear correlation between variables, with values ranging from-1 to 1. The calculation formula is as follows:

9 $$\:\text{R}=\frac{\sum\:_{\text{i}=1}^{\text{n}}\left({\text{X}}_{\text{i}}-{{\upsigma\:}}_{\text{X}}\right)\left({\text{Y}}_{\text{i}}-{{\upsigma\:}}_{\text{Y}}\right)}{\sqrt{\sum\:_{\text{i}=1}^{\text{n}}{\left({\text{X}}_{\text{i}}-{{\upsigma\:}}_{\text{X}}\right)}^{2}}\sqrt{\sum\:_{\text{i}=1}^{\text{n}}{\left({\text{Y}}_{\text{i}}-{{\upsigma\:}}_{\text{Y}}\right)}^{2}}}$$

where: R is the Pearson coefficient, X_i_ is the variable, Y_i_ is the target variable, $\:{{\upsigma\:}}_{\text{X}}$ is the mean of the feature variable, $\:{{\upsigma\:}}_{\text{Y}}$ is the mean of the target variable and n is the number of data samples.

**Fig. 6:**
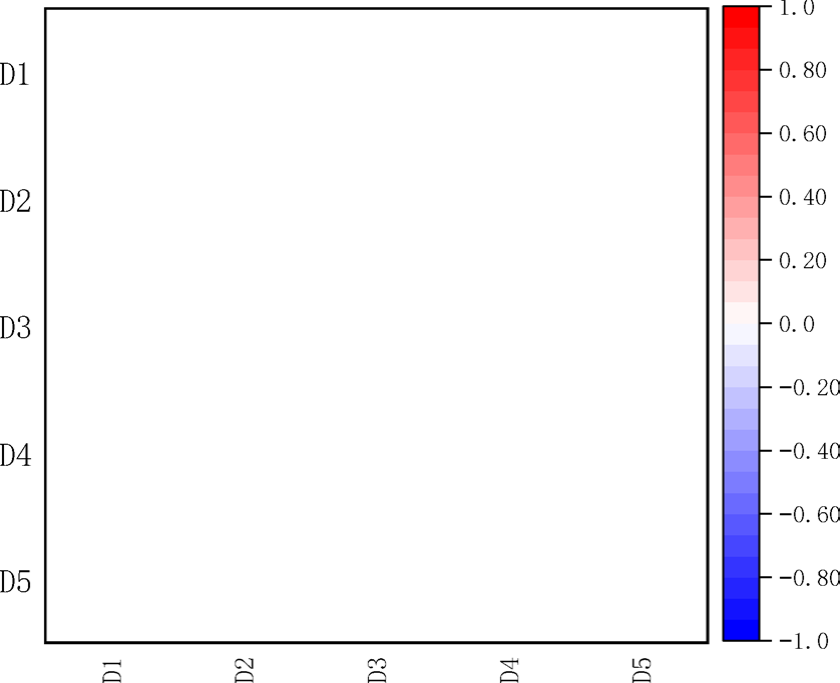
Heatmap of pearson correlation coefficients.

Figure 6 shows the feature correlation heat map of the collected data. By examining the correlation coefficients represented by the different colors of the blocks in the heatmap, we can determine the strength of the correlation between variables. If|R|< 0.4, the two variables are weakly correlated or uncorrelated; if 0.4≤|R|<0.8, the two variables are moderately correlated; and if 0.8≤|R|<1, the two variables are strongly correlated. Therefore, D2, D3, and D5 were selected as the feature factors influencing the gas concentration for the prediction.

### Comparison of univariate and multivariate gas concentration prediction

**Fig. 7:**
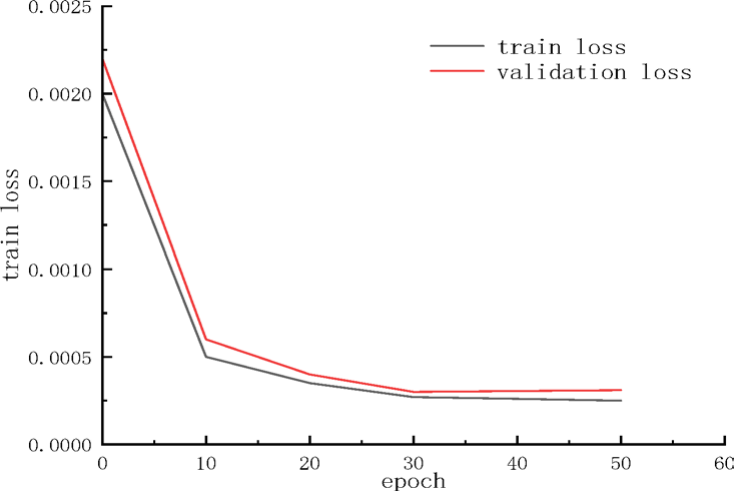
Training and validation loss curves of the SCBA model.

As shown in Fig. 7, the training and validation MSE curves maintain parallel convergence with a final gap of 0.0003 (< 5% of initial loss), indicating synchronized learning without divergence.

**Fig. 8:**
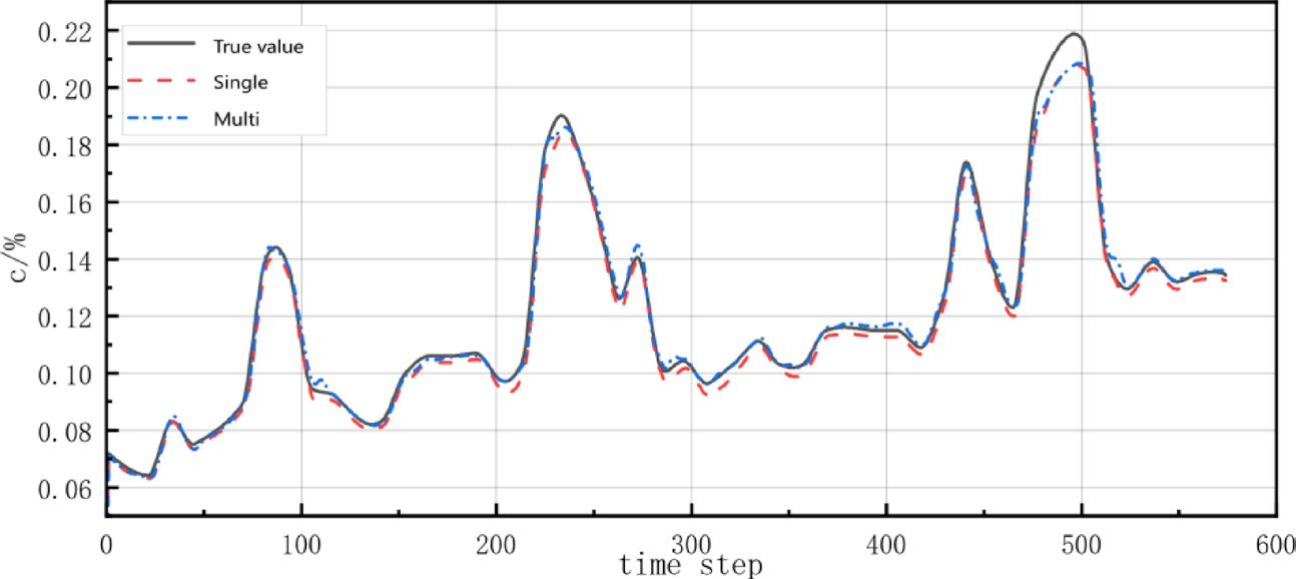
Univariate and multivariate gas concentration prediction curves.

To evaluate whether a multivariate gas prediction model could better reflect real-time environmental impacts on gas concentration compared to a univariate model, we compared two approaches: the univariate model using only historical gas concentration (D1) versus the multivariate model incorporating four time-series variables - gas concentration (D1), temperature (D2), wind speed (D3), and CO concentration (D5). The prediction results are shown in Fig. 8.

Figure 8 shows that the multivariate gas prediction closely matches the measured values in both trend and fluctuation patterns. For a rigorous comparison of these two prediction methods, we performed quantitative analysis using root mean square error (RMSE) and mean absolute percentage error (MAPE):

From Table 1, the RMSE of the multivariate model (0.028) was smaller than that of the univariate model (0.040), and the MAPE (0.159) was significantly lower than that of the univariate model (0.254). Compared to the univariate prediction, the multivariate model demonstrated improvements of 29.3% and 37.4% for RMSE and MAPE, respectively. These results indicate that the multivariate model outperformed the univariate model in both evaluation metrics, suggesting that incorporating multiple influencing factors (including environmental parameters) can better capture gas concentration variation patterns and enhance prediction performance.

**Table 1:** Univariate and multivariate predictive indicator values.

Parameter	RMSE	MAPE
Univariate	0.040	0.254
Multivariate	0.028	0.159

### Comparison of model prediction performance and ablation experiment analysis

#### Comparison of SCBA model with other models

To verify the prediction efficacy of the proposed SCBA (Sparrow Search Algorithm optimized CNN-BiLSTM-Attention) model for gas concentration prediction, this study compares the single-step prediction accuracy of four benchmark models: Attention-LSTM, SSA-LSTM-Attention, Transformer-LSTM, andCNN-BiLSTM-Attention.

**Fig. 9:**
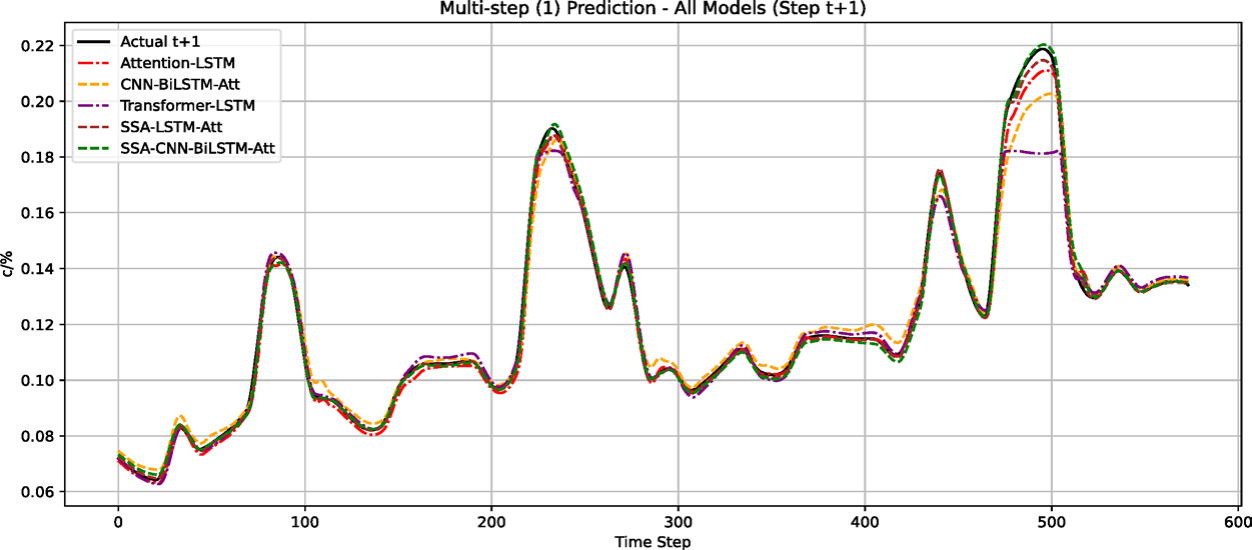
Comparison of gas prediction among different models.

Analysis of Fig. 9 reveals that the SCBA model’s predicted values demonstrate the closest agreement with measured values, indicating superior prediction performance among all evaluated models. For quantitative comparison, we employed two standard evaluation metrics: root mean square error (RMSE) and mean absolute percentage error (MAPE).

**Fig. 10:**
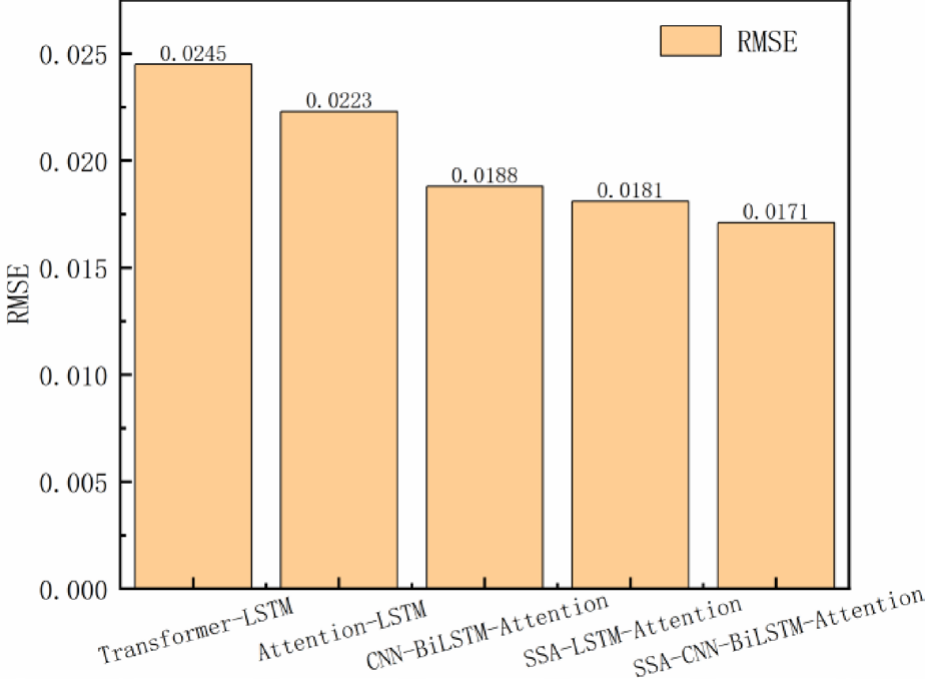
Comparison of RMSE predictions by each model.

**Fig. 11:**
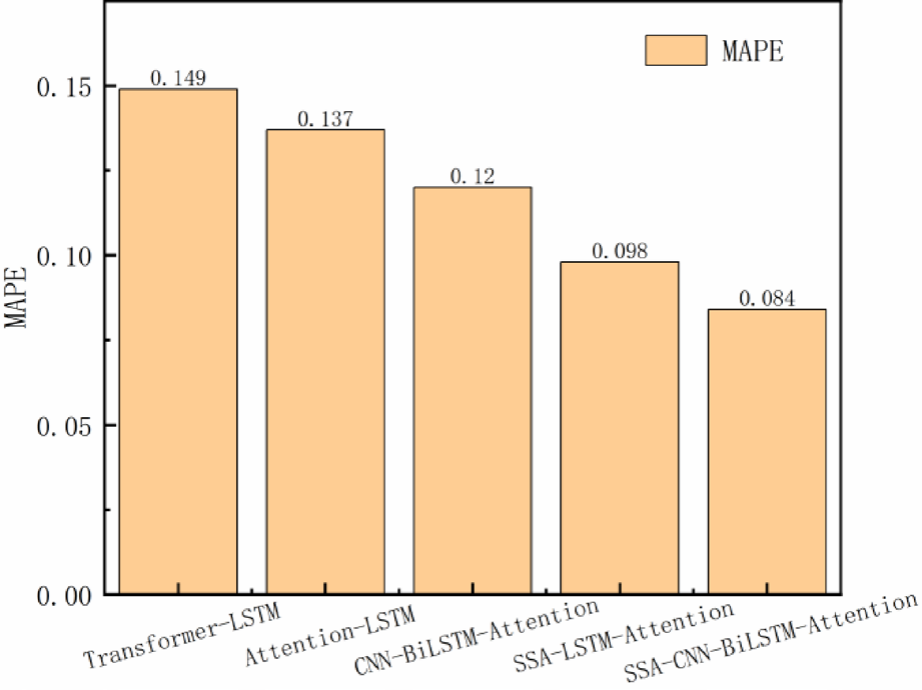
Comparison of MAPE predictions across different models.

The prediction errors of all models on the test set are systematically compared in Table 2 and visualized in Figs. 10 and 11. Quantitative analysis demonstrates that the SCBA model achieves superior performance with RMSE = 0.0171 and MAPE = 0.084, representing:23.3%, 4.4%, and 30.2% RMSE improvements over Attention-LSTM, SSA-LSTM-Attention, and Transformer-LSTM respectively; 38.7%, 14.3%, and 43.6% MAPE reductions compared to the same benchmark models.

**Table 2:** Predictive indicator values of different models.

Model	RMSE	MAPE
Attention-LSTM	0.0223	0.137
SSA-LSTM-Attention	0.0181	0.098
Transformer-LSTM	0.0245	0.149
CNN-BiLSTM-Attention	0.0188	0.12
SSA-CNN-BiLSTM-Attention	0.0171	0.084

### Contribution analysis of each module in the model based on ablation experiments

To systematically evaluate the contributions of key architectural components, the study conducted comprehensive ablation studies by sequentially removing individual modules (CNN, BiLSTM, and Attention) from the complete SCBA framework. This experimental design enables quantitative assessment of each module’s impact on overall prediction performance through comparative error analysis.

Analysis of the ablation study results (Table 3) reveals several key findings regarding model component contributions:

**Table 3:** Contribution error table of each module.

Model	RMSE	MAPE
SCBA	0.0171	0.084
Remove CNN	0.0191	0.13
Remove BiLSTM	0.0337	0.21
Remove Attention	0.0225	0.14
Only BiLSTM	0.0414	0.32

CNN Removal: Resulted in modest performance degradation (RMSE + 0.2%, MAPE + 4.6%), demonstrating its importance in extracting spatial features that capture local temperature-wind speed coupling relationships.

BiLSTM Removal: Caused the most significant performance decline, confirming its central role in modeling temporal dynamics of gas concentration variations.

Attention Mechanism Removal: Led to a 12.6% MAPE increase, particularly affecting prediction accuracy during abrupt concentration changes, thereby validating its critical weighting function for anomalous events.

Single-Module Configuration: The BiLSTM-only implementation yielded the highest errors, underscoring the necessity of multi-component integration for optimal performance.

These results collectively demonstrate that the synergistic interaction of CNN (spatial processing), BiLSTM (temporal modeling), and Attention (feature weighting) mechanisms is essential for achieving the model’s superior predictive capability.

### Comparison of prediction performance at different time steps

The purpose of gas concentration prediction is to identify the potential safety risks in advance. Short-term predictions at different time steps can help mine workers predict possible dangers before an accident occurs, thus optimizing the emergency response plans. The performance of different models or algorithms may vary in short- and long-term predictions, and comparing the prediction performance helps to identify the most suitable prediction model, ensuring the accuracy of gas concentration prediction and better prevention of safety risks. In practical predictions, it is essential to select an appropriate prediction step length to ensure the best prediction accuracy. Additionally, based on the actual requirements, a minimun escape time of 20 min should be reserved for mine workers. Therefore, this study analyzes predictions for 5, 10, 15, and 20 time steps.

**Fig. 12:**
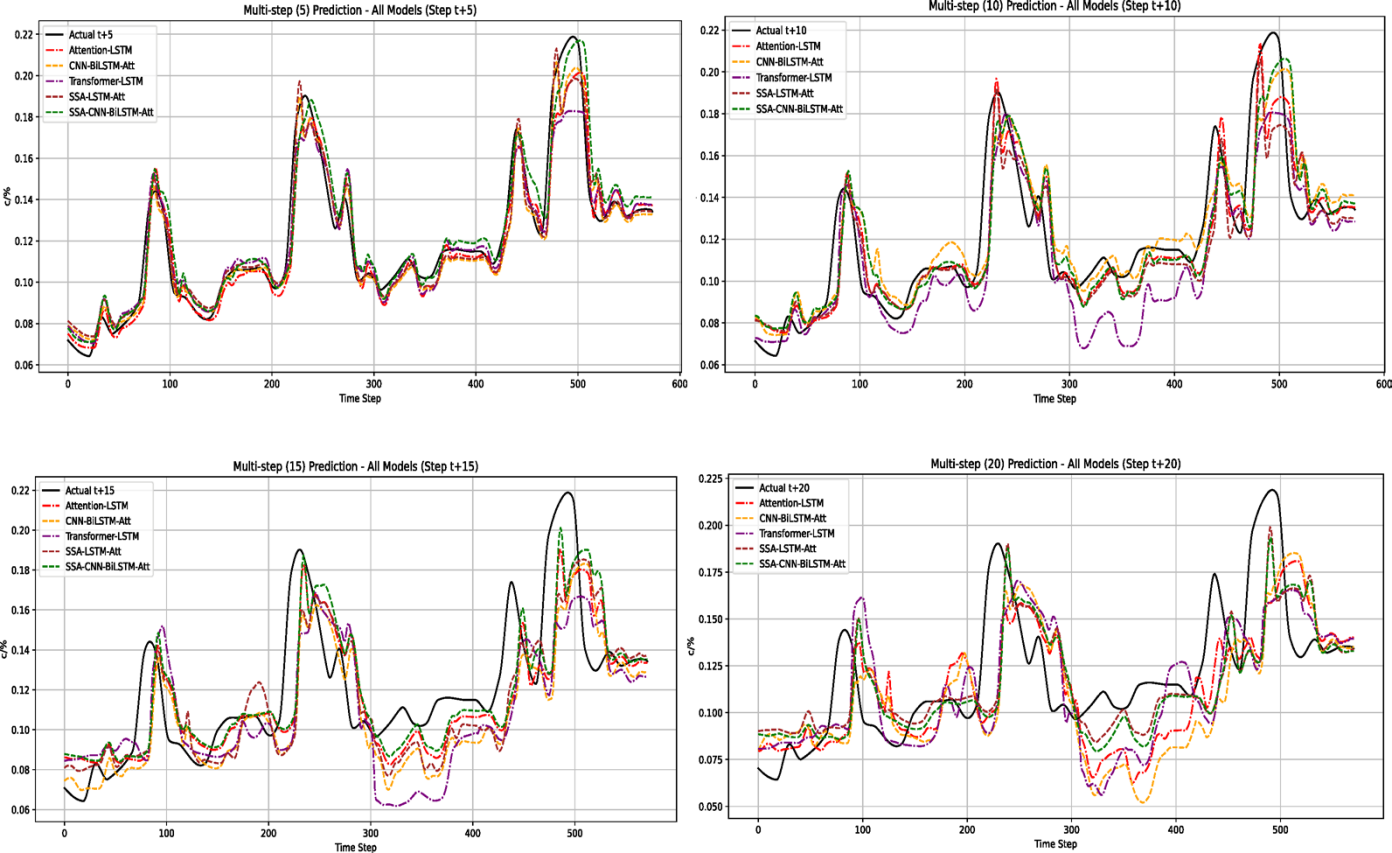
Gas prediction curves with different prediction step sizes.

The gas concentration prediction curves for different time steps are shown in Fig. 12. As the prediction step length increased, each model exhibited close fit to the gas concentration trend. However, the deviation between models varied across time steps.

**Fig. 13:**
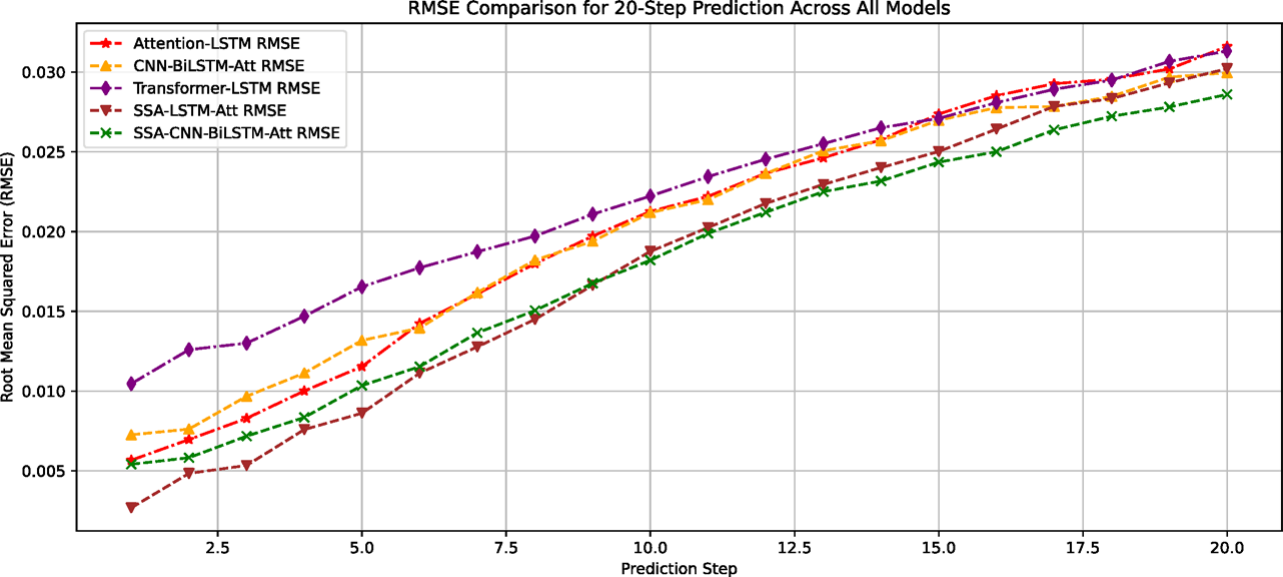
RMSE curves of each model at different prediction steps.

**Fig. 14:**
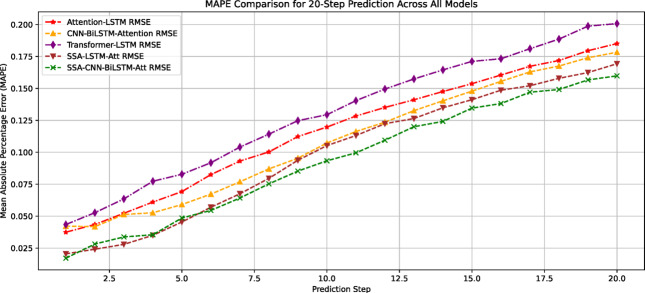
MAPE curves of each model under different prediction step sizes.

To analyze the impact of different step lengths on the prediction accuracy of different models more precisely, the root mean square error (RMSE) and mean absolute percentage error (MAPE) for each model at different step lengths were extracted, as shown in Figs. 13 and 14. The results indicate the following:

1. Overall prediction performance: The SCBA model demonstarted significant advantages, maintaining low error levels for both the RMSE and MAPE metrics. When the step length was 20, the RMSE of the model was 0.028, improving the prediction performance by 12.5%, 6.67%, 9.68%, and 6.67% compared with the other models. The MAPE was 0.159, which improved the performance of the other models by 14.52%, 11.17%, 20.90%, and 6.47%, respectively.
2. Variation in prediction accuracy with increasing step length: For the RMSE metric, in short-step predictions (before 10 steps), Model 1 (SSA-CNN-BiLSTM-Attention) had better prediction accuracy (RMSE = 0.002) than Model 2 (SSA-LSTM-Attention) (RMSE = 0.005). After the step length exceeded 10 steps, the error growth rate of Model 1 decreased, and the prediction error of Model 2 gradually exceeded that of Model 1. For the MAPE metric, in extremely short-term predictions with step lengths < 5, Model 2 maintained lower relative errors. When the step length exceeded five steps, model 1 exhibited stronger stability. This indicates that the prediction accuracy changed as the prediction step length increased. Therefore, in practical applications, an appropriate prediction model should be selected based on the number of sample prediction steps.
3. Overall trend: The error values of all models gradually increased with the prediction step length, indicating that as the prediction time span increased, the difficulty of prediction increased and accuracy gradually decreased. Comparing the RMSE and MAPE metrics of the different models, the Sparrow Optimization Algorithm-based CNN-BiLSTM-Attention model had the slowest error growth rate as the number of steps increased, with RMSE and MAPE increasing by 9.35%/step and 13.27%/step, respectively, further highlighting its superiority in short-term predictions. When the prediction step length was 20, the error values were the lowest compared with those of the other models. Therefore, this model can be used for anomaly analysis of the prediction results when the prediction step length is 20, thus reserving more escape time for mine workers.

## Conclusions

This study developed a novel CNN-BiLSTM-Attention framework (SCBA) for gas concentration prediction, integrating bidirectional LSTM temporal modeling with CNN-based spatial feature extraction and attention-based dynamic weighting. The hybrid architecture effectively addresses the challenge of multi-variable spatiotemporal feature fusion in underground mine environments. Experimental results demonstrate the SCBA model’s superior performance compared to attention-LSTM, SSA-LSTM-attention, and transformer-LSTM benchmarks, achieving 23.3%, 4.4%, and 30.2% RMSE improvements and 38.7%, 14.3%, and 43.6% MAPE reductions respectively. Ablation studies confirmed the critical contributions of each component, with BiLSTM emerging as the core temporal processor (most significant performance drop upon removal), CNN enabling crucial spatial feature extraction (4.6% MAPE increase when removed), and the attention mechanism proving essential for handling concentration anomalies (12.6% MAPE degradation without it).

The model’s operational value was particularly evident in 20-minute predictions (RMSE = 0.028), where it maintained 9.7–30.2% lower errors than alternatives while showing the slowest error growth rate (9.35% per step). This performance meets critical safety requirements by providing reliable early warnings within the minimum 20-minute evacuation window. However, several limitations should be noted:

(1) Data limitations.

The current model was trained exclusively on monitoring data from a single mining face in Shanxi (2,880 samples). Although data augmentation techniques were applied to enhance robustness, the model’s generalization capability across different geological conditions (e.g., coal seam thickness, gas occurrence patterns) and mining methods (fully-mechanized/blasting mining) may be limited, potentially leading to degraded prediction performance.

(2) Technical assumptions.

The model operates under the following potentially restrictive assumptions:

① The current 1-minute sampling interval may fail to capture sub-minute concentration fluctuations;

② Continuous sensor operation is assumed without accounting for equipment failures;

③ Static environment assumption: The model lacks dynamic adaptation to ventilation system adjustments or excavation progress changes.

Future work will focus on: (1) validating the model across diverse mining environments, (2) incorporating real-time equipment status monitoring to handle sensor failures, and (3) developing adaptive mechanisms for dynamic mining conditions. These enhancements will strengthen the model’s robustness for practical deployment while maintaining its demonstrated advantages in prediction accuracy and stability.

## Data Availability

Data is provided within the manuscript or supplementary information files, and other data sets generated during the current study are available from the corresponding author on reasonable request.
